# An Atypical Case of Licorice-Induced Pseudoaldosteronism Presenting With Decreased Urine Potassium Excretion in the Presence of Severe Hypokalemia in a Very Elderly Patient

**DOI:** 10.7759/cureus.76694

**Published:** 2024-12-31

**Authors:** Satoshi Kurisu, Hitoshi Fujiwara

**Affiliations:** 1 Department of Cardiology, National Hospital Organization (NHO) Hiroshima-Nishi Medical Center, Otake, JPN

**Keywords:** drug-induced hypokalemia, elderly people, licorice, resistent hypertension, urinary potassium

## Abstract

Most herbal medicines contain licorice, which may inhibit 11-beta-hydroxysteroid dehydrogenase type 2 (11βHSD2). When licorice inhibits 11βHSD2, accumulated cortisol binds excessively to the mineralocorticoid receptor (MR) instead of aldosterone, promoting sodium absorption and potassium excretion. This condition has been called pseudoaldosteronism due to its clinical manifestations resembling those of primary aldosteronism, producing excessive aldosterone. Primary aldosteronism and pseudoaldosteronism usually present with increased urine potassium excretion in the face of hypokalemia due to excessive MR activation by aldosterone and cortisol, respectively, the so-called renal potassium loss. Here, we report an atypical case of licorice-induced pseudoaldosteronism, which unexpectedly presented with decreased urine potassium excretion in the presence of severe hypokalemia in an elderly patient. A spot potassium-to-creatinine ratio was decreased to 4.5 mmol/g creatinine. A 24-hour urine collection also revealed decreased urine potassium excretion of 7.5 mmol/day in the presence of severe hypokalemia. These results on urine potassium were atypical for pseudoaldosteronism. Urine sodium excretion was also significantly reduced to 27.5 mmol/day, suggesting low sodium intake due to anorexia. Low sodium intake followed by low sodium delivery to the collecting duct was the crucial cause of decreased urine potassium excretion in pseudoaldosteronism. Clinicians need to understand the side effects of licorice and assess the risks and benefits associated with the use of licorice-containing drugs, especially in patients with risk factors for pseudoaldosteronism, such as advanced age or low body weight. Careful follow-up of blood pressure or serum potassium concentration is required to prevent the development of pseudoaldosteronism.

## Introduction

Herbal medicines have been widely used in Japan for various symptoms or conditions. Most herbal medicines contain licorice, which may inhibit 11-beta-hydroxysteroid dehydrogenase type 2 (11βHSD2). This enzyme is essentially responsible for decomposing cortisol to inactive cortisone, allowing aldosterone to bind mineralocorticoid receptors (MR) in the distal nephron [1-5]. When licorice inhibits 11βHSD2, accumulated cortisol binds excessively to MR instead of aldosterone, promoting sodium absorption and potassium excretion [5]. Since Conn et al. first reported this condition, it has been called pseudoaldosteronism due to its clinical manifestations resembling those of primary aldosteronism producing excessive aldosterone [6]. A recent study using the Japanese Adverse Drug Event Report database showed that shakuyakukanzoto (42.9%) was the most frequently suspected drug, followed by yokukansan (22.4%) and rikkunshito (5.7%), among 210 cases of pseudoaldosteronism [1].

Primary aldosteronism and pseudoaldosteronism typically manifest with increased urine potassium excretion in the face of hypokalemia due to excessive MR activation by aldosterone and cortisol, respectively, a phenomenon referred to as renal potassium loss [7]. In patients with hypokalemia, urine potassium excretion of more than 15 mmol/day in a 24-hour urine collection directly indicates inappropriate renal potassium loss [8]. However, this approach is difficult to use in outpatient or emergency care. A spot potassium-to-creatinine ratio of over 13 mmol/g creatinine is an alternative indication of inappropriate renal potassium loss if a 24-hour urine collection is not feasible [8].

Here, we report an atypical case of licorice-induced pseudoaldosteronism, which unexpectedly presented with decreased urine potassium excretion in the presence of severe hypokalemia in an elderly patient.

## Case presentation

A 90-year-old man with hypertension who had taken amlodipine (10 mg/day) and irbesartan (100 mg/day) for 10 years presented to his primary care clinic with a one-week history of anorexia and difficulty walking. He had a history of cerebral infarction and was taking prasugrel (3.75 mg/day) and pravastatin (10 mg/day). The patient also reported a weight gain of 5 kg with edema in both lower extremities. Laboratory tests showed an increased brain natriuretic peptide of 146.8 pg/mL. He was referred to our hospital for further cardiac evaluation after six days.

**Figure 1:**
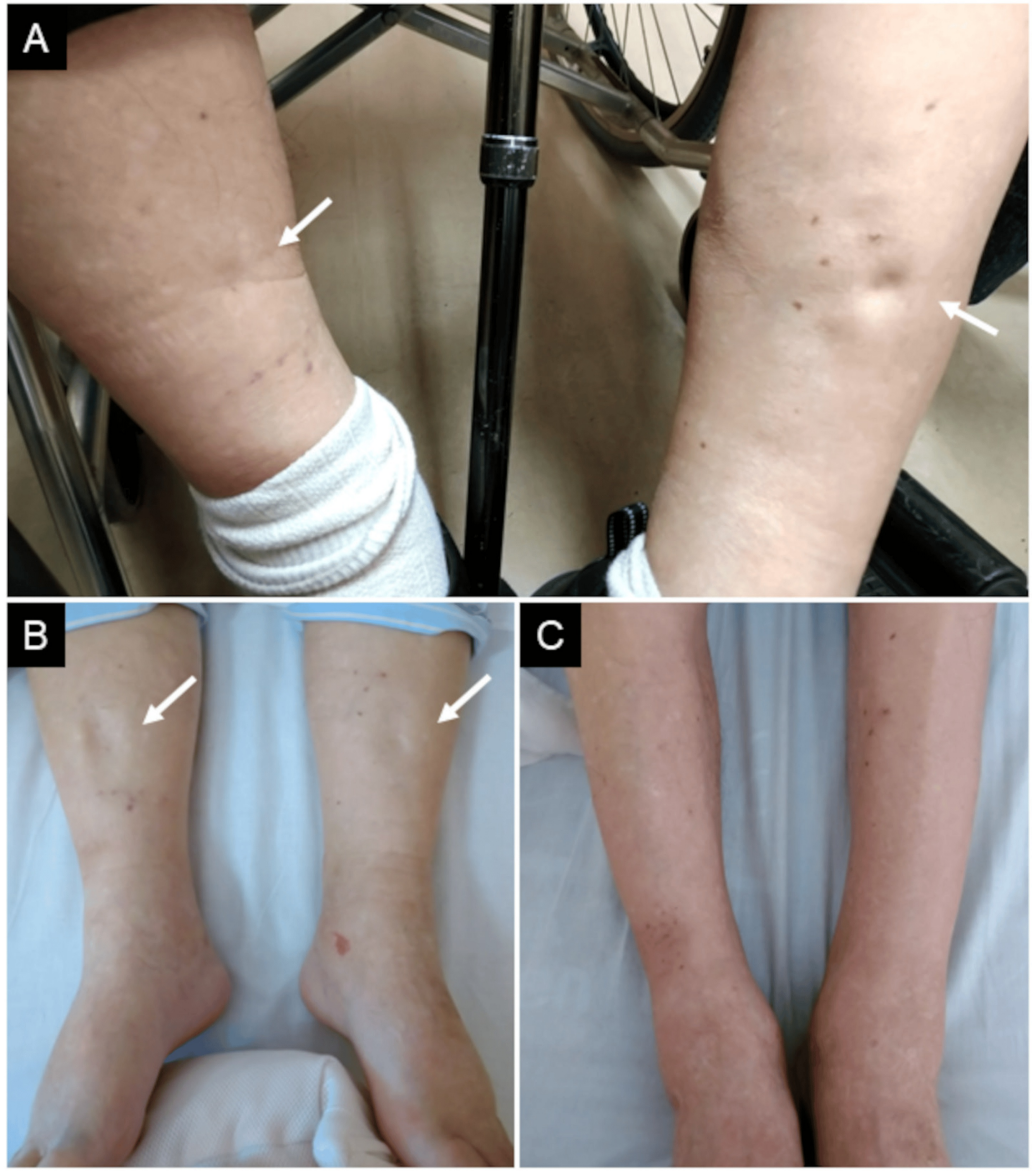
Both lower extremities before and after the treatment At initial presentation, significant pitting edema was noted in both lower extremities (A-B, arrows). On the 14th day, his pitting edema in the lower extremities disappeared (C).

On physical examination, his body weight was 74 kg; blood pressure was 130/78 mmHg; pulse rate was 70 bpm; and oxygen saturation was 96%. There were no audible murmurs. Significant pitting edema was noted in both lower extremities (Figures 1A, 1B). Muscle weakness prevented the patient from standing up independently. He was admitted for these conditions.

Laboratory tests revealed mild anemia with hemoglobin of 10.7 g/dL, severe hypokalemia with potassium of 1.91 mmol/L, and metabolic alkalosis with pH of 7.542 (Table 1). Serum magnesium concentration was normal at 0.99 mmol/L. Creatine kinase increased to 1,509 U/L. Based on these results, his symptom of difficulty walking was attributed to hypokalemic myopathy. The patient had taken the angiotensin II receptor antagonist irbesartan, which increases plasma renin activity [9]. Nevertheless, plasma renin activity was suppressed (0.4 ng/mL/hour), and plasma aldosterone concentration was low (< 4.0 pg/mL).

**Table 1:** Laboratory data

Variable	Day 1	Day 9	Day 17	Reference Range
Blood count				
White blood cell count (/µL)	6.9 × 10^3^	-	5.0 × 10^3^	3.3–8.6 × 10^3^
Red blood cell count (/µL)	3.86 × 10^6^	-	4.32 × 10^6^	4.35–5.55 × 10^6^
Hemoglobin (g/dL)	10.7	-	12.0	13.7–16.8
Hematocrit (%)	32.9	-	37.7	34.9–45.1
Platelet count (/µL)	241 × 10^3^	-	283 × 10^3^	158–348 × 10^3^
Blood chemistry				
Total bilirubin (mg/dL)	1.48	-	-	0.4–1.5
Aspartate aminotransferase (U/L)	46	-	-	13–30
Alanine aminotransferase (U/L)	19	-	-	10–42
Lactate dehydrogenase (U/L)	308	-	-	124–222
Creatine phosphokinase (U/L)	1,509	595	130	59–248
Total protein (g/dL)	6.3	-	-	6.6–8.1
Albumin (g/dL)	3.2	-	-	4.1–5.1
Blood urea nitrogen (mg/dL)	6.5	8.2	13.8	8–20
Creatinine (mg/dL)	1.17	1.01	1.18	0.65–1.07
Estimated glomerular filtration rate (mL/minute/1.73 m^2^)	44.9	52.7	50.6	-
Sodium (mmol/L)	141	143	143	138–145
Potassium (mmol/L)	1.91	2.57	4.67	3.6–4.8
Chloride (mmol/L)	96	101	109	101–108
Magnesium (mmol/L)	0.99	-	-	0.74–1.07
C-reactive protein (mg/dL)	0.19	-	-	< 0.14
Brain natriuretic peptide (pg/mL)	78.7	-	44.0	< 18.4
Plasma renin activity (ng/mL/hour)	0.4	-	-	-
Plasma aldosterone concentration (pg/mL)	< 0.4	-	-	4.0–82.1
Cortisol (μg/dL)	15.6	-	-	7.07–19.6
Thyroid-stimulating hormone (μIU/mL)	1.32	-	-	0.61–4.23
Free thyroxine (ng/dL)	1.46	-	-	0.7–1.48
Venous gas analysis				
pH	7.542	7.485	7.396	-
HCO_3_^-^ (mmol/L)	35.0	36.0	29.5	-
Base excess (mmol/L)	11.5	11.3	4.3	
24-hour urinalysis				
Sodium (mmol/day)	27.5	-	-	-
Potassium (mmol/day)	7.5	-	-	-
Chloride (mmol/day)	30	-	-	-
Urine volume (mL)	1250	-	-	-
Creatinine (mg/dL)	41.9	-	-	-
Spot urinalysis				
Sodium (mmol/L)	< 20	78	148	-
Potassium (mmol/L)	8	3	7	-
Chloride (mmol/L)	< 20	56	92	-
Potassium-to-creatinine ratio (mmol/g creatinine)	4.5	6.6	19.9	-

**Figure 2:**
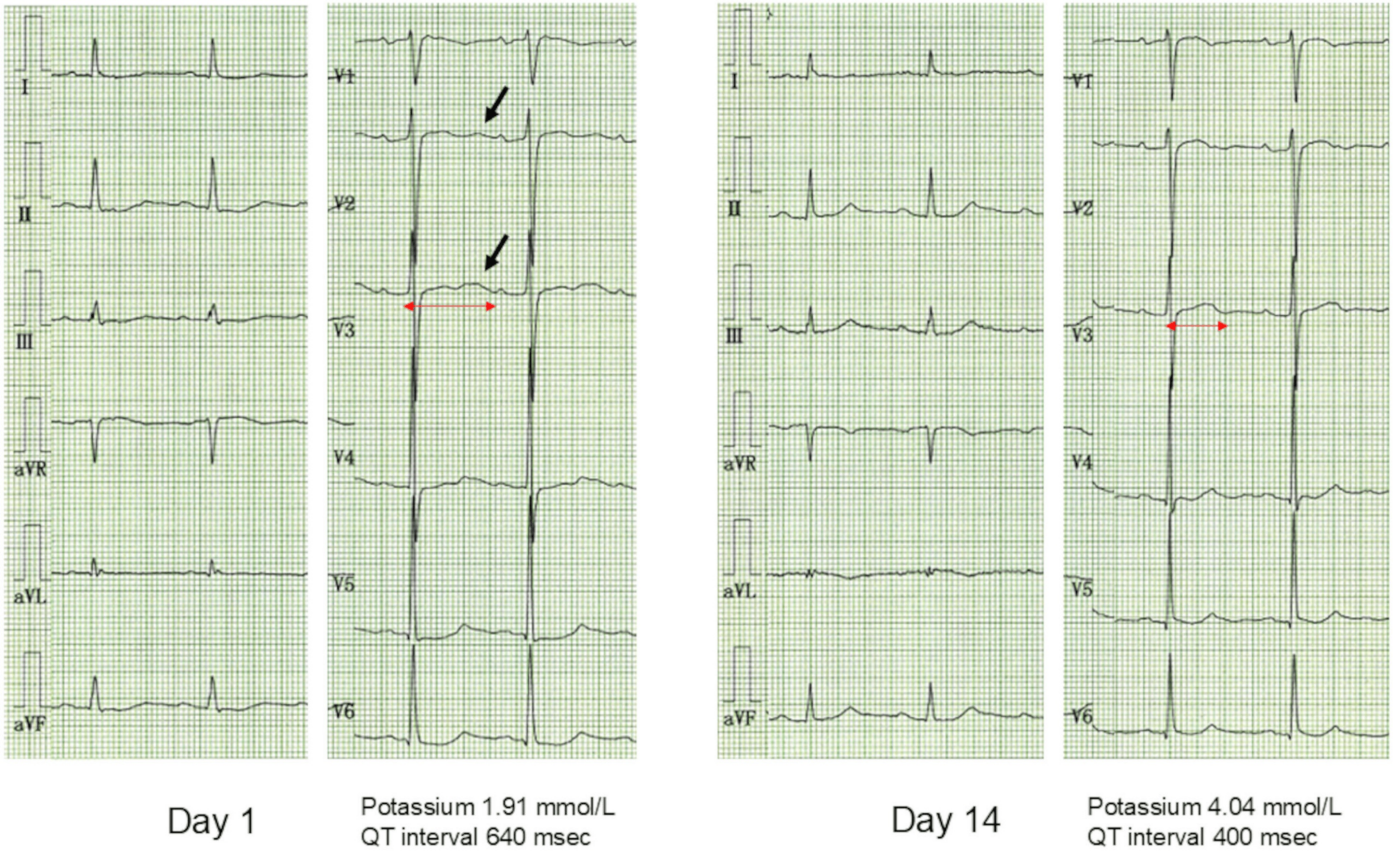
Initial and follow-up electrocardiograms An initial electrocardiogram (ECG) showed ST-segment depressions in leads II, aV_F_, and V_4-6_. Prominent U waves were seen in leads V_2-3_ (A, arrows). QT interval was prolonged to 640 msec. On the 14th day, ST-segment depressions and U waves disappeared (B). The QT interval returned to normal at 400 msec.

An initial electrocardiogram (ECG) showed ST-segment depressions in leads II, aV_F_, and V_4-6_. Prominent U waves were seen in leads V_2-3_ (Figure 2A, arrows). QT interval was prolonged to 640 msec. These ECG findings were consistent with those of severe hypokalemia.

**Figure 3:**
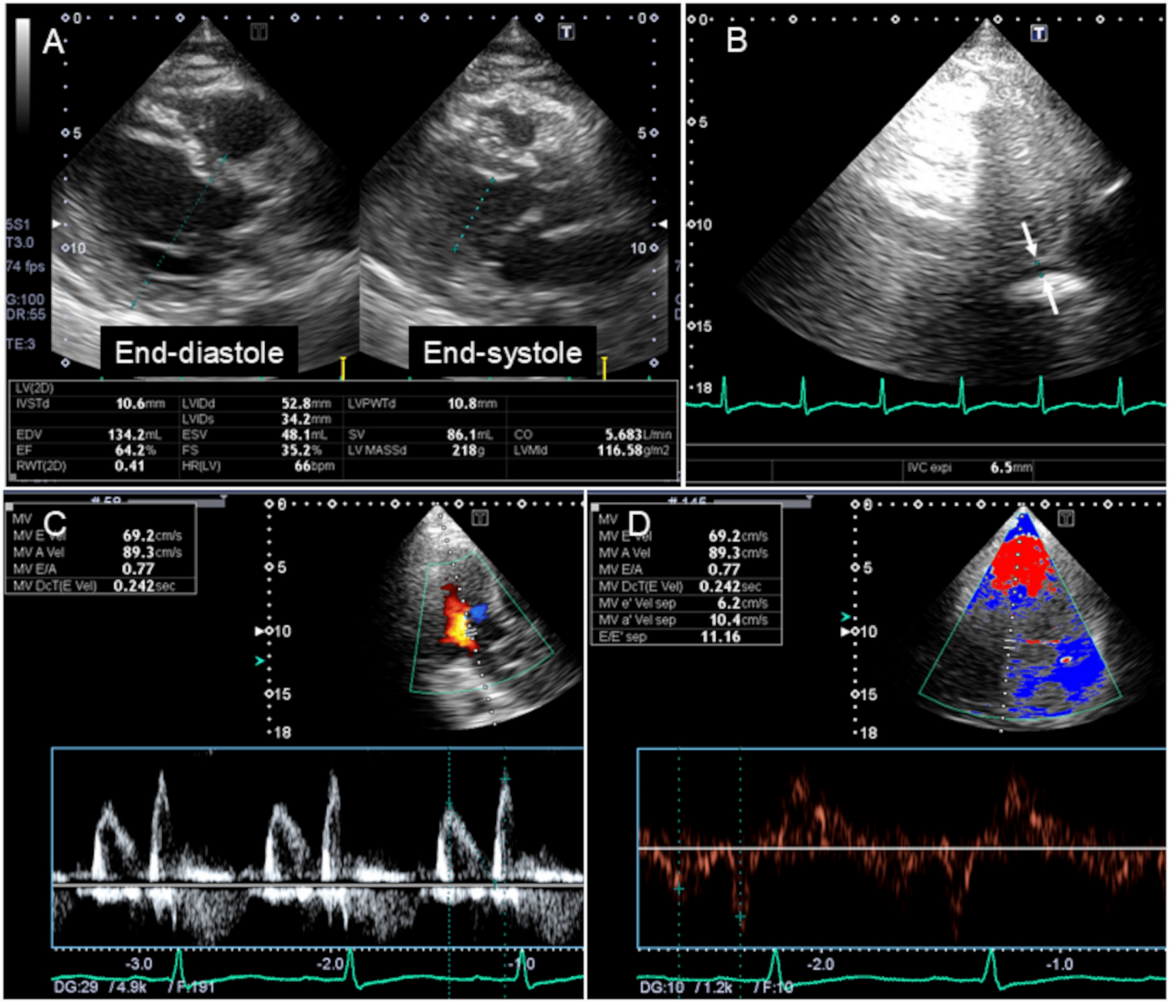
Transthoracic echocardiographic images A transthoracic echocardiogram showed normal left ventricular systolic function with an ejection fraction of 67% (A). The diameter of the inferior vena cava at end-expiration was 7 mm (B, arrows), and it collapsed completely at end-inspiration, suggesting decreased intravascular volume due to anorexia. Doppler studies showed an early to late diastolic mitral flow velocity ratio of 0.77 (C) and an early diastolic mitral annular velocity of 6.2 cm/second (D).

A transthoracic echocardiogram showed normal left ventricular systolic function with an ejection fraction of 67% (Figure 3A). The diameter of the inferior vena cava at end-expiration was 7 mm (Figure 3B, arrows), and it collapsed completely at end-inspiration, suggesting decreased intravascular volume due to anorexia [10]. No significant valvular heart diseases were seen. Doppler studies showed an early (E) to late diastolic mitral flow velocity ratio of 0.77 (Figure 3C) and early diastolic mitral annular velocity (e’) of 6.2 cm/second (Figure 3D). The three parameters recommended in the 2016 diastolic function guideline [11] were as follows: average E/e’ of 11.0, tricuspid regurgitation flow velocity of 2.7 m/second, and left atrial volume index of 35 mL/m^2^. Based on these results, the patient was diagnosed with grade I diastolic dysfunction.

Repeated careful history-taking revealed that he had been taking the herbal medicine shakuyakukanzoto (2.5 g/day) from another clinic for muscle clamps for five years. His daily dose of licorice from the herbal medicine was 2.0 g. His medical history and laboratory data, such as suppressed plasma renin activity and low plasma aldosterone concentration, strongly suggested licorice-induced pseudoaldosteronism. A spot potassium-to-creatinine ratio decreased to 4.5 mmol/g creatinine. A 24-hour urine collection also revealed decreased urine potassium excretion of 7.5 mmol/day in the presence of severe hypokalemia. These results on urine potassium were atypical for pseudoaldosteronism (Table 1). Urine sodium excretion was also significantly reduced to 27.5 mmol/day, suggesting low sodium intake due to anorexia.

**Figure 4:**
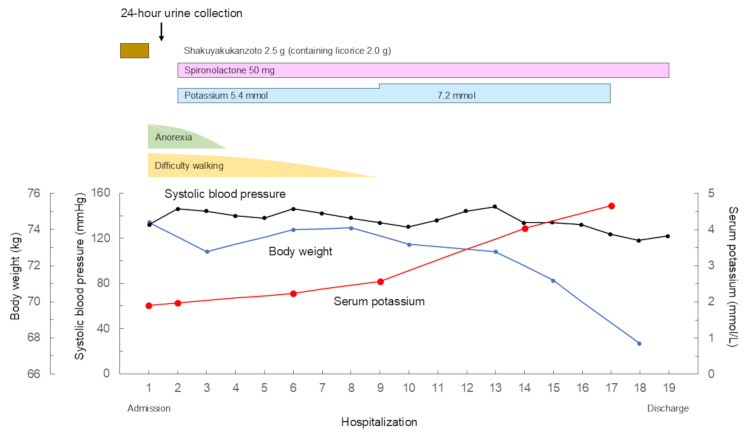
Clinical course The herbal medicine shakuyakukanzoto was discontinued immediately after admission, and oral administration of potassium and spironolactone (50 mg/day) was started. His symptoms, such as anorexia and difficulty walking, gradually improved, and serum potassium concentration returned to 4.04 mmol/L on the 14th day.

The herbal medicine shakuyakukanzoto was discontinued immediately after admission, and oral administration of potassium and spironolactone (50 mg/day) was started (Figure 4). His symptoms, such as anorexia and difficulty walking, gradually improved, and serum potassium concentration returned to 4.04 mmol/L on the 14th day.

Corresponding to the normalization of serum potassium concentration, ST-segment depressions and U waves disappeared. The QT interval returned to normal at 400 msec (Figure 2B). The pitting edema in his lower extremities disappeared (Figure 1C), and his body weight returned to 68 kg. Oral potassium supplementation was stopped before discharge. Spironolactone treatment was continued for another week after discharge. The patient was discharged on his own on the 19th day.

## Discussion

In this report, we showed an atypical case of licorice-induced pseudoaldosteronism presenting with decreased urine potassium excretion in the presence of severe hypokalemia in a very elderly patient.

Herbal medicines are more frequently prescribed in clinical practice in Japan than in Western countries. Most herbal medicines contain licorice. Licorice may inhibit 11βHSD2, leading to the accumulation of cortisol [1-5]. Cortisol acts on MR and promotes sodium absorption and potassium excretion in the collecting duct. Uneda et al. recently identified female sex, older age, low body weight, diuretics usage, hypertension, and dementia as pseudoaldosteronism-related factors using a data-mining approach [1]. Based on previous clinical studies, Mantani et al. examined the relationship between pseudoaldosteronism incidence and daily licorice dose [12]. They demonstrated that the incidences with 2 g/day, 4 g/day, and 6 g/day were 1.7%, 3.3%, and 11.1%, respectively. Their results suggested a dose-dependent trend toward pseudoaldosteronism incidence.

In pseudoaldosteronism, licorice inhibits 11βHSD2, resulting in low plasma renin activity and low plasma aldosterone concentration. Limited data exist concerning the recovery of the renin-aldosterone system after the withdrawal of licorice. Epstein et al. reported that the renin-aldosterone system returned to normal within two to four months in four subjects who had ingested 25 to 200 g/day of licorice for more than six months [13].

In the present case, the patient was a very elderly man with normal body weight, and his daily dose of licorice was only 2.0 g. Several studies have shown a decrease in 11βHSD2 activity with age [14]. This may explain why the patient newly developed pseudoaldosteronism at the advanced age of 90 years, despite the same amount of licorice. Serum potassium concentration returned to normal approximately two weeks after the discontinuation of shakuyakukanzoto. Spironolactone treatment was continued for another week after discharge. Our case again emphasizes that even a small amount of licorice can cause pseudoaldosteronism. Clinicians need to understand the side effects of licorice and assess the risks and benefits associated with the use of licorice-containing drugs, especially in patients with risk factors for pseudoaldosteronism, such as advanced age or low body weight. Careful follow-up of blood pressure or serum potassium concentration is required to prevent the development of pseudoaldosteronism.

The most interesting finding of this case was that urine potassium excretion was decreased to 7.5 mmol/day despite the condition of pseudoaldosteronism. Homeostatic mechanisms maintain the serum potassium concentration within a narrow range despite variable potassium intake. In healthy subjects, potassium excretion is mediated mainly by the kidney. Potassium filtered through the glomerulus is almost completely absorbed before reaching the collecting duct. Almost all urine potassium is secreted from the collecting duct [15]. It has been clearly established that serum potassium concentration can modulate aldosterone biosynthesis. There is a feedback mechanism for aldosterone: a decrease in serum potassium concentration inhibits the secretion of aldosterone [7], thereby leading to decreased urine potassium excretion. In the present case, the patient had a one-week history of anorexia as one of the symptoms of pseudoaldosteronism, as confirmed by a collapsed inferior vena cava. Therefore, both sodium intake and potassium intake must have been reduced. In the condition of pseudoaldosteronism, low potassium intake does not work in the above-mentioned feedback mechanism for aldosterone because accumulated cortisol continues to act on MR instead of aldosterone.

**Figure 5:**
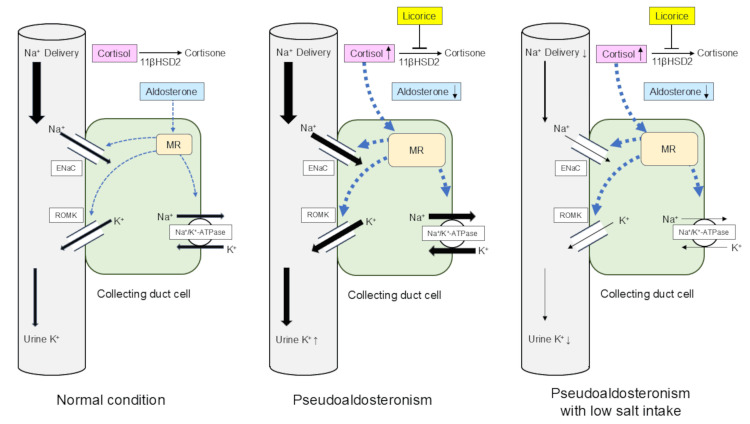
Schematic diagrams showing the effects of licorice and salt restriction In normal conditions, aldosterone promotes potassium excretion through its effects on Na+/K+-ATPase and epithelial sodium and potassium channels in the collecting duct (A). When pseudoaldosteronism develops, accumulated cortisol excessively activates MR, promoting sodium absorption and potassium excretion. As a result, urine potassium excretion in the collecting duct increases (B). When sodium intake becomes reduced in pseudoaldosteronism, sodium filtered through the glomerulus is almost absorbed before reaching the collecting duct, resulting in low sodium delivery to the collecting duct (C). Image created by the authors. MR: mineralcorticoid receptor; 11βHSD2: 11-beta-hydroxysteroid dehydrogenase type 2; ENaC: epithelial sodium channel; ROMK: renal outer medullary potassium channel

In the present case, decreased urine potassium excretion in pseudoaldosteronism can be explained by low sodium intake, confirmed by decreased urine sodium excretion of 27.5 mmol/day. In normal conditions, aldosterone promotes potassium excretion through its effects on Na+/K+-ATPase and epithelial sodium and potassium channels in the collecting duct (Figure 5A) [7]. When pseudoaldosteronism develops, accumulated cortisol excessively activates MR, promoting sodium absorption and potassium excretion. As a result, urine potassium excretion in the collecting duct increases (Figure 5B). When sodium intake becomes reduced in pseudoaldosteronism, sodium filtered through the glomerulus is almost absorbed before reaching the collecting duct, resulting in low sodium delivery to the collecting duct (Figure 5C). In fact, potassium excretion in the collecting duct is regulated by sodium delivery and flow rate as well as the activity of MR [16]. In the present case, it is suggested that sodium absorption in the collecting duct became reduced due to low sodium delivery despite excessive activity of MR, consequently leading to decreased potassium excretion in the collecting duct. Low sodium intake followed by low sodium delivery to the collecting duct was the crucial cause of decreased urine potassium excretion in pseudoaldosteronism.

There have been few reports regarding the effect of sodium depletion on urine potassium excretion in the setting of primary aldosteronism or pseudoaldosteronism. Shioji et al. reported a case of primary aldosteronism with a salt-free diet for nine days without changing potassium intake [17]. The patient's urine sodium excretion decreased from 124.7 mmol/day to 13.7 mmol/day, and urine potassium excretion decreased from 34.3 mmol/day to 11.9 mmol/day after six days of salt restriction. Both urine sodium excretion and potassium excretion increased again after salt repletion. Takakuwa et al. examined the effect of sodium restriction on urine potassium excretion in six patients with aldosterone-producing adenoma [18]. The mean urine potassium excretion was 45 mmol/day on a normal salt diet (10-12 g/day) and 37 mmol/day on a low salt diet (2-4 g/day). Zhou et al. divided 50 patients with idiopathic hyperaldosteronism into a normal sodium diet group and a low sodium diet group and showed urine potassium excretion in the two groups [19]. The mean urine potassium excretion was 45.4 mmol/day on a normal sodium diet (100 mmol/day) and 33.0 mmol/day on a low sodium diet (50 mmol/day). According to these reports, a strict sodium restriction appears to be necessary to induce urine potassium excretion of less than 15 mmol/day. The results of these reports support our explanation for the relationship between sodium intake and urine potassium excretion.

## Conclusions

In conclusion, we encountered an atypical case of licorice-induced pseudoaldosteronism presenting with decreased urine potassium excretion in the presence of severe hypokalemia. Low sodium intake followed by low sodium delivery to the collecting duct was the crucial cause of decreased urine potassium excretion in pseudoaldosteronism. Our case again emphasizes that even a small amount of licorice can cause pseudoaldosteronism. Clinicians need to understand the side effects of licorice and assess the risks and benefits associated with the use of licorice-containing drugs, especially in patients with risk factors for pseudoaldosteronism, such as advanced age or low body weight. Careful follow-up of blood pressure or serum potassium concentration is required to prevent the development of pseudoaldosteronism.
